# Are HOMA-IR and HOMA-B good predictors for diabetes and pre-diabetes subtypes?

**DOI:** 10.1186/s12902-023-01291-9

**Published:** 2023-02-14

**Authors:** Davood Khalili, Marjan Khayamzadeh, Karim Kohansal, Noushin Sadat Ahanchi, Mitra Hasheminia, Farzad Hadaegh, Maryam Tohidi, Fereidoun Azizi, Ali Siamak Habibi-Moeini

**Affiliations:** 1 Prevention of Metabolic Disorders Research Center, Research Institute for Endocrine Sciences, Shahid Beheshti University of Medical Sciences, Tehran, 1985717413 Iran; 2 Department of Biostatistics and Epidemiology, Research Institute for Endocrine Sciences, Shahid Beheshti University of Medical Sciences, Tehran, Iran; 3 Endocrine Research Center, Research Institute for Endocrine Sciences, Shahid Beheshti University of Medical Sciences, Tehran, Iran

**Keywords:** Prediabetes, Impaired glucose tolerance, Impaired fasting glucose, HOMA-IR, HOMA-B, Diabetes, Prediabetic state

## Abstract

**Background:**

To investigate the association between the Homeostasis Model Assessment of Insulin Resistance (HOMA-IR) and Homeostasis Model Assessment of Beta-cell function (HOMA-B) with the incidence of diabetes and pre-diabetes subtypes.

**Methods:**

A total of 3101 normoglycemic people aged 20–70 years were included in the 6-year follow-up study. Multinomial logistic regression was used to calculate the incidence possibility of isolated Impaired Fasting Glucose (iIFG), isolated Impaired Glucose Tolerance (iIGT), Combined impaired fasting glucose & impaired glucose tolerance (CGI), and Diabetes Mellitus (DM) per standard deviation (SD) increment in HOMA-IR and HOMA-B in the crude and multivariable model.

**Results:**

In the multivariate model, an increase in one SD change in HOMA-IR was associated with a 43, 42, 75, and 92% increased risk of iIFG, iIGT, CGI, and DM, respectively. There was a positive correlation between the increase in HOMA-B and the incidence of iIGT; however, after adjusting the results for metabolic syndrome components, it was inversely correlated with the incidence of iIFG [Odds Ratio = 0.86(0.75–0.99)].

**Conclusions:**

HOMA-IR is positively correlated with diabetes and pre-diabetes subtypes’ incidence, and HOMA-B is inversely correlated with the incidence of iIFG but positively correlated with iIGT incidence. However, none of these alone is a good criterion for predicting diabetes and pre-diabetes.

**Supplementary Information:**

The online version contains supplementary material available at 10.1186/s12902-023-01291-9.

## Introduction

Type 2 diabetes is a growing disease worldwide, imposing a severe burden on society along with its complications [1–4]. In Iran, the incidence rate of type 2 diabetes in the urban population over 20 years is more than 1 % annually [5]. The incidence rate of pre-diabetes is significantly higher and estimated to be more than 4 % each year, indicating a very high prevalence of diabetes soon [6].

Pre-diabetes is associated with a high risk of micro and macrovascular complications [7–10]. As a result, early detection of people susceptible to pre-diabetes, lifestyle modifications, and effective medications is necessary to prevent developing pre-diabetes and its complications in these people [4, 11]. 30-year results of the Da Qing Diabetes Prevention Outcome Study showed that lifestyle intervention in people with pre-diabetes could reduce 40% development of diabetes and delay its onset by 3·96 years [12]. Insulin resistance and dysfunction of pancreatic beta cells are important factors in the pathophysiology of diabetes and pre-diabetes, contributing to different degrees among pre-diabetic subgroups according to different races and ethnicities [13–16]. There are various methods for measuring insulin resistance and insulin secretion dysfunction. Hyperinsulinaemic euglycemic clamp and hyperglycemic clamp are the best methods; however, their application has been limited due to the difficulty of preformation, time-consuming implementation, and high costs [17]. The most common method used in epidemiological studies is HOMA-B and HOMA-IR formulas. There is a high correlation between the results of this method and the results of the clamp method [18–20]. A cut-off point seems to be a good diagnostic measure for different populations and races [19].

Studies conducted in different communities have used different ligation methods or HOMA to investigate the association between insulin resistance or impaired insulin secretion and pre-diabetes subtypes and also have produced different results. On the other hand, these studies have been mainly cross-sectional that are inapplicable for predicting the incidence of pre-diabetes. Moreover, no separate study has determined the appropriate cut-off points for HOMA-IR and HOMA-B to predict the incidence of diabetes and pre-diabetes in our community. Consequently, this study was conducted on Tehran Lipid and Glucose Study (TLGS) population to prospectively answer if there is a prospective association between “insulin resistance or insulin secretion disorder” and “diabetes and pre-diabetes subtypes” in our society.

## Methods

### Study population

**Fig. 1:**
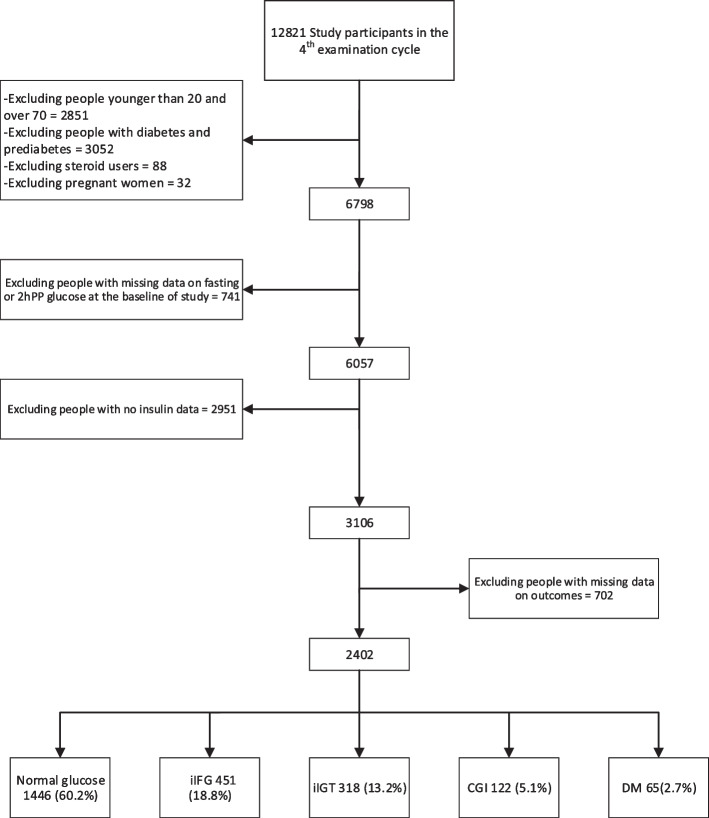
Study participants flowchart

This study was performed on the Tehran Lipid and Glucose Study population, representing the Tehran population. The methodology of TLGS has already been published [5]. This study has been performed on 12,819 individuals who participated in the fourth examination cycle of the study, of which we exclude people younger than 20 and over 70, those with diabetes or pre-diabetes, pregnant women, steroid users, and those with missing data on fasting blood sugar (FBS) and two-hour blood sugar (2hpp BS) at baseline. After exclusion, a total of 3101 people remained who were followed up to the sixth examination cycle in triennial intervals (mean period of 6 years). During follow-up, people with missing data on outcomes were excluded, and finally, 2399 subjects remained in the study. Participants were followed up for the incidence of pre-diabetes or diabetes or the last follow-up time, and each happened first (Fig. 1).

### Clinical and laboratory measurements

After obtaining written informed consent, all participants were referred to trained physicians. Demographic data, disease records, family history of non-communicable diseases, smoking habits, physical activity assessment, and physical examination, including anthropometric measurements, were collected and performed by physicians.

Blood samples were taken after 12–14 hours of overnight fasting, between 7 and 9 am, and centrifuged within 30–45 minutes of collection. Plasma glucose was measured 2 hours after receiving 75 g of oral glucose using an enzymatic calorimetry method with glucose oxidase technique on the day of sample collection. The coefficients of variation (CV) for inter and intra-test at the beginning and follow-up of the TLGS were 2.2% for glucose. The serum was used to measure fasting insulin (FI) too. After collecting the samples, insulin was measured by electrochemiluminescence immunoassay method using the Roche Diagnostics kits and a Roche/Hitachi Cobas e-411 analyzer (GmbH, Manheim, Germany). Lyophilized quality control material (Lyphochek Immunoassay Plus Control, Bio-Rad Laboratories) was used to monitor the accuracy of assays. Intra- and inter-assay CV were 1.2% & 3.5%, respectively.

### Definitions

Diabetic patients included those with FBS ≥ 126 mg/dl or BS2hpp ≥ 200 mg/dl at the beginning or during the study or those who received antidiabetic therapy. Subjects with BS2hpp < 140 mg/dl and FBS < 100 mg/dl were considered as free of diabetes, isolated impaired fasting glucose (iIFG) was considered in cases with FBS between 100 and 125 mg/dl and BS2hpp < 140 mg/dl, isolated impaired glucose tolerance (iIGT) included FBS < 100 mg/dl and BS2hpp between 140 and 199 mg/dl, combined impaired fasting glucose & impaired glucose tolerance (CGI) was defined as FBS between 100 and 125 mg/dl and BS2hpp between 140 and 199 mg/dl [21, 22]. Pre-defined cut-offs from the previous statement from the Iranian National Committee of Obesity report were used to define binary metabolic syndrome variables [23]. HOMA-IR was calculated using the formula FBS(mmol/L)*FI(Mu/ml)/22.5 [24, 25], and HOMA-B was calculated using the formula 20*FI(Mu/ml)/[FBS(mmol/L)-3.5] [19, 26].

### Statistical analysis

Initially, the normality distribution of the variables was assessed graphically using Q-Q plots, and the baseline characteristics of the participants were described. Mean (SD) and median (IQR) were used for continuous variables with normal and skewed distributions, respectively. Also, frequency (%) was described for the categorical variables. Depending on the variable type, Pearson’s Chi-square test, ANOVA, and Kruskal Wallis tests were used to compare baseline indices in the normal group, iIFG, iIGT, CGI, and DM. The Cubic Spline Models using three knots based on quartiles of the variables were used to evaluate the linear relationship between HOMA-IR and HOMA-B with the incidence of pre-diabetes/diabetes. Multinomial Logistic Regression model was used to evaluate the association between changes in HOMA-IR and HOMA-B with incidences of iIFG, iIGT, CGI, and DM. Since the association between HOMA-IR/HOMA-B and pre-diabetes/diabetes incidence was linear, odds ratio (ORs) and 95% confidence intervals (95% CIs) for exposure variables per one SD change were reported.

Three models were designed for analysis. Model (1): Crude model, Model (2): Adjusted for gender and age, smoking, education level, and family history of diabetes, Model (3a): Adjusted for model 2 in addition to metabolic syndrome variables including waist circumference, blood pressure, triglyceride, and HDL, as binary variables, model (3b): Adjusted for model 2 in addition to metabolic syndrome variables including waist circumference, systolic & diastolic blood pressure, triglyceride, and HDL as continuous variables.

ROC curve analysis was used to determine the possible relationship between HOMA-B and HOMA-IR with the risk of diabetes and pre-diabetes over 6 years. ROC curve analysis for HOMA-B and HOMA-IR was repeated in pre-diabetes subtypes (iIFG, iIGT). SPSS version 20 and STATA version 14 software were used for these analyses. The results with a *P*-value of less than 0.05 were considered significant.

The ethics committee of Shahid Beheshti University of Medical Sciences approved this study.

## Results

As shown in Fig. 1, of the 2399 individuals followed up for 6 years, 2.7% had DM, 5.1% had CGI, 13.2% had iIGT, and 18.8% had iIFG as the first event.

Table 1 shows the baseline characteristics of study participants at the beginning of the fourth examination cycle. This table compared subjects in 5 events of the normal group, iIFG, iIGT, CGI, and DM group. Significant differences were observed between groups in almost all variables.

**Table 1:** Baseline characteristics of study participants in different categories of outcomes

		Total	Normal glucose	iIFG	iIGT	CGI	DM	*P*-Value
Number		2399 (100%)	1443 (60.2%)	318 (13.2%)	451 (18.8%)	122 (5.1%)	65 (2.7%)	
Age (years)		42.6 (11.36)	40.96 (10.62)	48.5 (11.70)	44.9 (10.38)	48 (11.22)	43.65 (12.25)	< 0.001
Female (%)		1477 (61.7%)	948 (65.6%)	193 (60.7%)	229 (50.1%)	63 (52.1%)	44 (67.7%)	< 0.001
Education (%) (a)	0	379 (15.9%)	188 (13%)	83 (26.1%)	80 (17.7%)	20 (16.5%)	10 (15.4%)	< 0.001
	1	1379 (57.5%)	831 (57.5%)	176 (55.3%)	259 (57.4%)	71 (58.7%)	41 (63.1%)	–
	2	641 (26.5%)	426 (29.5%)	59 (18.6%)	112 (24.8%)	30 (24.8%)	14 (21.5%)	–
Smoking (%) (b)	0	1796 (74.9%)	1114 (77.1%)	231 (72.6%)	304 (67.4%)	99 (81.8%)	48 (73.8%)	< 0.001
	1	163 (6.8%)	71 (4.9%)	33 (10.4%)	43 (9.5%)	10 (8.3%)	6 (9.2%)	–
	2	440 (18.3%)	258 (17.9%)	54 (17%)	104 (23.1%)	12 (9.9%)	11 (16.9%)	–
Familial history of type 2 DM (%)		240 (10%)	133 (9.2%)	39 (12.3%)	49 (10.9%)	8 (6.6%)	11 (16.9%)	0.08
Body mass index (kg/m^2^)		27.5 (4.56)	27 (4.3)	28.64 (4.33)	28.1 (4.7)	29.6 (4.38)	30.55 (5.37)	< 0.001
Waist circumstance (cm)		93 (11)	90.8 (10.8)	95.5 (11)	97 (10)	98 (10.1)	98 (13.2)	< 0.001
Systolic Blood Pressure (mmHg)		112.83 (15.82)	110.51 (14.133)	118.8 (16.79)	114 (15.7)	121.8 (17.8)	120.7 (21)	< 0.001
Diastolic blood pressure (mmHg)		75.67 (10.94)	74.65 (10.51)	78 (10.79)	76.5 (11.38)	80.4 (10.4)	79.9 (12.4)	< 0.001
FBS (mmol/dl)		5.00 (0.31)	4.95 (0.30)	5.03 (0.29)	5.18 (0.26)	5.14 (0.30)	5.04 (0.33)	< 0.001
2hpp BS (mmol/dl)		5.32 (1.09)	5.12 (1.04)	5.93 (1.06)	5.44 (1.09)	5.96 (1.00)	5.86 (1.20)	< 0.001
HDL-C (mmol/dl)		1.25 (0.30)	1.28 (0.31)	1.24 (0.30)	1.19 (0.27)	1.17 (0.26)	11.19 (0.22)	< 0.001
Triglyceride (mmol/dl)		1.27 (0.90–1.81)	1.17 (0.86–1.65)	1.43 (1.03–1.99)	1.47 (1.05–1.99)	1.62 (1.20–2.35)	1.53 (1.15–2.12)	< 0.001
Total cholesterol (mmol/dl)		4.86 (0.87)	4.78 (0.92)	5.05 (0.95)	5.12 (0.85)	5.14 (0.86)	5.03 (0.86)	< 0.001
Fasting serum insulin (micro U/ml)		7.64 (5.10–50.69)	7.32 (5.32–9.93)	8.06 (5.8–11.36)	8.09 (5.6–11.6)	8.36 (6.58–12.67)	10.19 (6.7–13.9)	< 0.001
HOMA-IR		1.69 (1.20–2.40)	1.61 (1.15–2.21)	1.78 (1.27–2.55)	1.89 (1.28–2.72)	1.94 (1.48–2.83)	2.43 (1.44–3.08)	< 0.001
HOMA-B		103.7 (64–151)	102.6 (74–141)	108.4 (75–149)	98.4 (78–140)	107.2 (77–164)	127.6 (88–204)	< 0.001

a) Education: 0 = illiterate/primary; 1 = below diploma; 2 = higher than diploma

b) Smoking: 0 = Nonsmoker; 1 = past smoker; 2 = current smoker

**Fig. 2:**
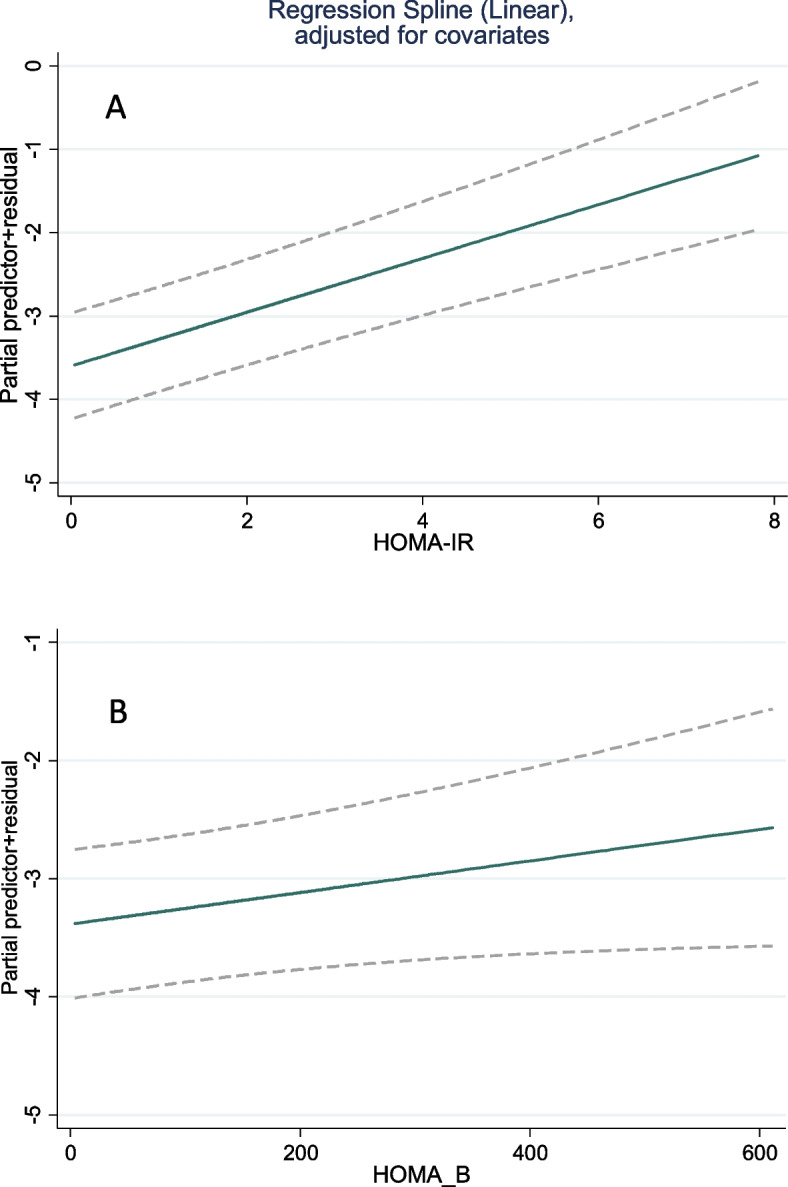
Relationship between **A** HOMA-IR and pre-diabetes/diabetes incidence **B** HOMA-B and pre-diabetes/diabetes incidence

The results of the cubic spline are reflected in Fig. 2, which indicates the linearity of the association between the HOMA-IR and HOMA-B with the incidence of outcomes.

As shown in Table 2 for the crude model, a unit increase in SD of HOMA-IR was associated with increased odds of iIFG by 1.36, iIGT by 1.28, CGI by 1.57, and DM by 1.83 times. After adjusting for confounding variables, there was a slight increase in OR in Model 2 and a slight decrease in Model 3, but the differences were not significant. Also, in the crude model, one SD increase in HOMA-B was associated with a significantly increased odds of DM by 1.61, iIGT by 1.15, and CGI by 1.2 folds, but no significant relationship was observed with iIFG. After adjusting for confounding variables in models 2 and 3, there was no significant increase in risks of iIFG and iIGT. Only in model 3b a one-unit increase of SD of HOMA-B was associated with a reduction in the risk of iIFG incidence.

**Table 2:** Odds ratios of incidence of diabetes and pre-diabetes for HOMA-IR and HOMA-B

	iIFG	iIGT	CGI	DM
HOMA-IR				
Model-1	1.36 (1.22–1.51)	1.28 (1.13–1.45)	1.57 (1.35–1.84)	1.83 (1.52–2.19)
Model-2	1.43 (1.28–1.59)	1.42 (1.25–1.61)	1.75 (1.48–2.06)	1.92 (1.59–2.32)
Model-3a	1.38 (1.22–1.55)	1.33 (1.16–1.52)	1.5 (1.25–1.81)	1.70 (1.38–2.10)
Model-3b	1.31 (1.16–1.48)	1.28 (1.11–1.48)	1.42 (1.17–1.72)	1.54 (1.24–1.92)
HOMA-B				
Model-1	0.94 (0.83–1.05)	1.15 (1.02–1.29)	1.20 (1.01–1.42)	1.61 (1.36–1.90)
Model-2	1.01 (0.90–1.14)	1.31 (1.16–1.48)	1.37 (1.15–1.62)	1.72 (1.44–2.05)
Model-3a	0.92 (0.81–1.05)	1.20 (1.05–1.37)	1.13 (0.93–1.38)	1.53 (1.26–1.85)
Model-3b	0.86 (0.75–0.99)	1.17 (1.02–1.33)	1.08 (0.88–1.32)	1.41 (1.15–1.72)

Model (1): crude model, Model (2): Adjusted for gender and age, smoking, level of education, and family history of type 2 diabetes, Model (3a): Adjusted for model 2 in addition to metabolic syndrome variables including waist circumference, blood pressure, triglyceride, and HDL, as binary variables, model (3b): Adjusted for model 2 in addition to metabolic syndrome variables including waist circumference, systolic & diastolic blood pressure, triglyceride, and HDL as continuous variables

The main missing variable in our dataset was the insulin data (other missing variables were < 5%). The supplementary data represent comparisons between subjects with missing and non-missing data for insulin (Table S1) and subjects who followed and did not follow (Table S2). As there were some differences, we also did a sensitivity analysis considering multiple imputations (with the assumption of missing at random, i.e., the missing values depend on the measured variables), and the results changed negligibly (Table S3).

**Fig. 3:**
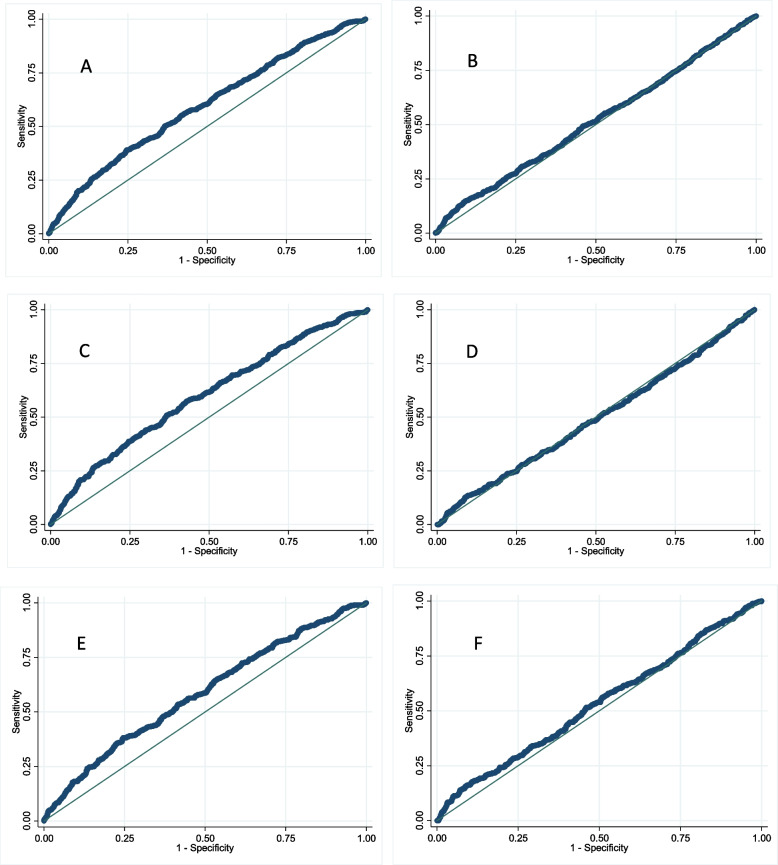
ROC diagram for the association between **A** HOMA-IR and **B** HOMA-B with the incidence of pre-diabetes/diabetes **C** HOMA-IR and incidence of iIFG, **D** HOMA-B and incidence of iIFG, **E** HOMA-IR and incidence of iIGT, **F** HOMA-B and incidence of iIGT, during 6 years. ROC curves show discrimination between iIFG and iIGT with normoglycemia

The area under the HOMA-IR curve (AUC) for pre-diabetes/diabetes was 59% (CI: 57–62%), and the best cut-off point based on Youden’s index was 2.22 (with 39% sensitivity and 76% specificity); also, the value for HOMA-B was 51% (CI: 49–54%) (Fig. 3) which was not statistically significant. The AUC of HOMA-IR and HOMA-B for iIFG (58 and 48%, respectively) and iIGT (56 and 52%, respectively) are also shown in Fig. 3, where the results are not meaningfully different from the analysis for the combined outcome.

## Discussion

This cohort study showed that in normoglycemic individuals increase in HOMA-IR has an association with an increased incidence of iIFG, iIGT, CGI, and DM during a six-year follow-up. It also showed increasing in HOMA-B is associated with an increased incidence of iIGT, CGI, and DM and decreased incidence of iIFG after adjusting for the variables of metabolic syndrome.

This study found a linear relationship between HOMA-IR and HOMA-B with the incidence of pre-diabetes. Similarly, in a study by Salami et al., who evaluated the incidence of pre-diabetes based on the HOMA-IR index in different quartiles, this risk was higher in the fourth quartile than the previous ones in almost all cases, including DM, CGI, iIGT, and iIFG [20].

There was no significant difference in the association between increased HOMA-IR and the incidence of pre-diabetes subtypes. As expected, OR in the diabetes group was significantly higher than either iIFG or iIGT. Also, there was no significant difference after adjusting for other variables.

In the case of HOMA-B, its increase has been associated with an increased incidence of DM, CGI, and iIGT, but in the case of iIFG, a significant reduction of incidence was only observed in model 3b (after adjustment for metabolic syndrome variables). In iIGT, peripheral resistance (muscle and fat) and relative insulin secretion impairment seem to play an essential role in developing pre-diabetes and diabetes, while in the case of iIFG, insulin resistance and hepatic glucose production have the primary role. Hence, comparing these two processes in iIFG and iIGT is not sufficiently accurate, especially using the HOMA-B formula, in which only fasting blood glucose and insulin are used. On the other hand, HOMA-B followed an increasing trend for 3 to 4 years before the onset of diabetes and then decreased until the incidence of diabetes [27]; thus, the effects of HOMA-B changes on the incidence of pre-diabetes can be affected by the time interval until the desired outcome in the future.

Also, in a study by Derakhshan et al., beta-cell dysfunction or low HOMA-B (HOMA-B < 25 percentile) was associated with increased risk of iIFG in a multivariate model [H.R. = 1.37(1.03–1.81) in men, and HR = 1.36(1.02–1.80) in women] which is in line with our findings. This study further showed that increased HOMA-IR was associated with increased HR in all four groups, which is in line with the results of our study, too [28]. A 2009 prospective study in Denmark to determine the association between insulin sensitivity and insulin secretion with the development of normoglycemic individuals to IFG or IGT reported that liver insulin resistance and subsequent dysfunction of beta cells in the secretion of insulin are the main factors in the progression of normal blood glucose to iIFG during 5 years, while the reduction of whole-body sensitivity and consequently insulin secretion dysfunction was reported as the major factors leading to iIGT [21]. It should be noted that, in this study, OGTT was performed, HOMA-S and ISI (Insulin Sensitivity Index) were used to assess insulin sensitivity, and EPIR (Early Phase Insulin Release) and DI (Disposition Index) were used to measure insulin secretion [29].

Too many studies investigated the association of HOMA-IR and HOMA-B with IFG and IGT. However, most of these were cross-sectional studies and evaluated this association concurrently with the occurrence of phenotypes, making it impossible to compare their results with the findings of this study. In this study, increasing HOMA-B per SD resulted in a reduced incidence of iIFG and an increased incidence of iIGT, CGI, and DM; Thus, AUC was not an appropriate criterion for predicting the development of pre-diabetes. On the other hand, HOMA-IR does not produce an acceptable AUC and cut-off point, so HOMA-IR is not a good index for predicting pre-diabetes. None of the ROC diagrams of HOMA-IR and HOMA-B, which were prepared separately for iIFG and iIGT, resulted in appropriate AUCs. In a study by Dr. Ghassemi et al., the HOMA-IR cut-off point for diagnosis of diabetes was 2.17 (50% sensitivity and 76.7% specificity) in women and 1.85% (75.9% sensitivity and 58.3% specificity) in men [19]. Compared with the results of Dr. Ghassemi’s study for diabetes, the cut-off point of our study seems to be inappropriate for predicting the incidence of pre-diabetes because the HOMA-IR cut-off point for pre-diabetes is expected to be lower than that for diabetes. Also, in a cross-sectional study by Baek et al., the reported cut-off point of HOMA-IR for detecting dysglycemia based on FBS and HbA1C (equal to 1.6) was lower than that obtained in our study [30]. Given that the HOMA-IR formula only includes fasting glucose and insulin, mainly related to insulin resistance in the liver, it seems complicated to obtain a single HOMA-IR cut-off point for all pre-diabetic subjects.

Since insulin levels and glucose concentrations are the only measurements needed to calculate HOMA-IR and HOMA-B, they have become the most widely used surrogate indicators, providing valuable insights into insulin resistance, b-cell function, and glucose metabolism [26, 31, 32]. However, in our findings, these were not good criteria for predicting the incidence of diabetes/pre-diabetes, emphasizing that HOMA should not be considered exclusively in the framework of clinical practice. Using other clinical features of subjects or indices such as triglyceride-glucose index [33], which has been shown that are important in predicting metabolic syndrome alongside HOMA-B/HOMA-IR, could help to decide more precisely for individuals concerning the prediction of pre-diabetes/diabetes in the future. Nonetheless, significant differences in insulin levels have been reported between different populations and ethnicities [34], and further research may be needed to re-evaluate our findings in other populations.

This study has some limitations and benefits. The main limitation of this study is the inability to use more precise methods to measure insulin resistance and its secretion (such as the clamp technique). Other limitations include the lack of information on HbA1C and missing participants during the study. Although the technique used in this study for measuring insulin (Electrochemical Luminescence) is currently one of the most precise methods, since there is no reference method for measuring insulin and the results of insulin measurement by different methods can vary up to 2 times, the method of insulin measurement should be considered while using the results [35].

The advantages of this study are as follows: It is the only study that evaluated the HOMA-IR cut-off point to predict the incidence of pre-diabetes. This study was performed in a high sample size and is the only study that prospectively investigates the association between changes in HOMA-B and HOMA-IR with the incidence of diabetes and pre-diabetes subtypes. In this study, the data analysis was performed using a multinomial logistic regression method, suitable for investigating the concurrent relationship between HOMA-B and HOMA-IR with several outcomes (iIFG, iIGT, CGI, and DM). This method seems to be more appropriate for investigating this relationship than the COX method used in the previous study.

In conclusion**,** this study showed that increased HOMA-IR in normoglycemic individuals is associated with increased incidence of pre-diabetes in both iIFG and iIGT subtypes, while increased HOMA-B leads to a higher risk of developing iIGT and lower risk of iIFG. There was no significant difference between the incidence of iIGT and iIFG with increased HOMA-IR. Based on the results of this study, it seems that cut-off points of HOMA-IR and HOMA-B are not suitable criteria for predicting the incidence of pre-diabetes.

## Supplementary Information

**Additional file 1: Table S1.** Baseline characteristics of study subjects with missing and non-missing data for insulin. **Table S2.** Baseline characteristics of study subjects subjects who were followed and not followed. **Table S3.** Odds ratios of incidence of different study outcomes for HOMA-IR and HOMA-B in multiply imputed data.

## Data Availability

The datasets used and/or analyzed during the current study are available from the corresponding author upon reasonable request.

## References

[1] Yip WC, Sequeira IR, Plank LD, Poppitt SD (2017). Prevalence of pre-diabetes across ethnicities: a review of impaired fasting glucose (IFG) and impaired glucose tolerance (IGT) for classification of dysglycaemia. Nutrients..

[2] Zheng Y, Ley SH, Hu FB (2018). Global aetiology and epidemiology of type 2 diabetes mellitus and its complications. Nat Rev Endocrinol.

[3] Chen L, Magliano DJ, Zimmet PZ (2012). The worldwide epidemiology of type 2 diabetes mellitus—present and future perspectives. Nat Rev Endocrinol.

[4] Perreault L, Færch K (2014). Approaching pre-diabetes. J Diabetes Complications.

[5] Harati H, Hadaegh F, Saadat N, Azizi F (2009). Population-based incidence of type 2 diabetes and its associated risk factors: results from a six-year cohort study in Iran. BMC Public Health.

[6] Hadaegh F, Derakhshan A, Zafari N, Khalili D, Mirbolouk M, Saadat N (2017). Pre-diabetes tsunami: incidence rates and risk factors of pre-diabetes and its different phenotypes over 9 years of follow-up. Diabet Med.

[7] Saydah SH, Loria CM, Eberhardt MS, Brancati FL (2001). Subclinical states of glucose intolerance and risk of death in the US. Diabetes Care.

[8] Brannick B, Dagogo-Jack S (2018). Prediabetes and cardiovascular disease: pathophysiology and interventions for prevention and risk reduction. Endocrinol Metab Clin.

[9] Brannick B, Wynn A, Dagogo-Jack S (2016). Prediabetes as a toxic environment for the initiation of microvascular and macrovascular complications. Exp Biol Med.

[10] Saydah SH, Miret M, Sung J, Varas C, Gause D, Brancati FL (2001). Postchallenge hyperglycemia and mortality in a national sample of US adults. Diabetes Care.

[11] Rhee SY, Woo J-T (2011). The prediabetic period: review of clinical aspects. Diabetes Metab J.

[12] Gong Q, Zhang P, Wang J, Ma J, An Y, Chen Y (2019). Morbidity and mortality after lifestyle intervention for people with impaired glucose tolerance: 30-year results of the Da Qing diabetes prevention outcome study. Lancet Diabetes Endocrinol.

[13] Kodama K, Tojjar D, Yamada S, Toda K, Patel CJ, Butte AJ (2013). Ethnic differences in the relationship between insulin sensitivity and insulin response: a systematic review and meta-analysis. Diabetes Care.

[14] Bando Y, Ushiogi Y, Okafuji K, Toya D, Tanaka N, Fujisawa M (2001). The relationship of fasting plasma glucose values and other variables to 2-h postload plasma glucose in Japanese subjects. Diabetes Care.

[15] Suzuki H, Fukushima M, Usami M, Ikeda M, Taniguchi A, Nakai Y (2003). Factors responsible for development from normal glucose tolerance to isolated postchallenge hyperglycemia. Diabetes Care.

[16] Aoyama-Sasabe S, Fukushima M, Xin X, Taniguchi A, Nakai Y, Mitsui R, et al. Insulin secretory defect and insulin resistance in isolated impaired fasting glucose and isolated impaired glucose tolerance. J Diabetes Res. 2016;2016(1298601):1–8. 10.1155/2016/1298601 PMC4693016 26788515

[17] Bloomgarden ZT (2006). Measures of insulin sensitivity. Clin Lab Med.

[18] Tm W, Levy J, Matthews D (2004). Use and abuse of HOMA modelling. Diabetes Care.

[19] Ghasemi A, Tohidi M, Derakhshan A, Hasheminia M, Azizi F, Hadaegh F (2015). Cut-off points of homeostasis model assessment of insulin resistance, beta-cell function, and fasting serum insulin to identify future type 2 diabetes: Tehran Lipid and Glucose Study. Acta Diabetol.

[20] Salami M, Hosseinpanah F, Azizi F (2006). Correlation of insulin resistance and impaired glucose metabolism in Tehranian adults: Tehran Lipid and Glucose Study. Iran J Endocrinol Metab.

[21] Unwin N, Shaw J, Zimmet P, Alberti K (2002). Impaired glucose tolerance and impaired fasting glycaemia: the current status on definition and intervention. Diabet Med.

[22] Association AD. Introduction: standards of medical care in diabetes—2020. Am Diabetes Assoc. 2020:S1–2. 10.2337/dc20-Sint 31862741

[23] Azizi F, Hadaegh F, Khalili D, Esteghamati A, Hossein PF, Delavari A (2010). Appropriate definition of metabolic syndrome among Iranian adults: report of the Iranian National Committee of Obesity.

[24] Muniyappa R, Lee S, Chen H, Quon MJ (2008). Current approaches for assessing insulin sensitivity and resistance in vivo: advantages, limitations, and appropriate usage. Am J Physiol-Endocrinol Metab.

[25] Meyer C, Pimenta W, Woerle HJ, Van Haeften T, Szoke E, Mitrakou A (2006). Different mechanisms for impaired fasting glucose and impaired postprandial glucose tolerance in humans. Diabetes Care.

[26] Song Y, Manson JE, Tinker L, Howard BV, Kuller LH, Nathan L (2007). Insulin sensitivity and insulin secretion determined by homeostasis model assessment and risk of diabetes in a multiethnic cohort of women: the Women’s Health Initiative Observational Study. Diabetes Care.

[27] Tabák AG, Jokela M, Akbaraly TN, Brunner EJ, Kivimäki M, Witte DR (2009). Trajectories of glycaemia, insulin sensitivity, and insulin secretion before diagnosis of type 2 diabetes: an analysis from the Whitehall II study. Lancet.

[28] Derakhshan A, Tohidi M, Arshi B, Khalili D, Azizi F, Hadaegh F (2015). Relationship of hyperinsulinaemia, insulin resistance and β-cell dysfunction with incident diabetes and pre-diabetes: the Tehran Lipid And Glucose Study. Diabet Med.

[29] Færch K, Vaag A, Holst JJ, Hansen T, Jørgensen T, Borch-Johnsen K (2009). Natural history of insulin sensitivity and insulin secretion in the progression from normal glucose tolerance to impaired fasting glycemia and impaired glucose tolerance: the Inter99 study. Diabetes Care.

[30] Baek JH, Kim H, Kim KY, Jung J (2018). Insulin resistance and the risk of diabetes and dysglycemia in Korean general adult population. Diabetes Metab J.

[31] Abbasi F, Okeke Q, Reaven GM (2014). Evaluation of fasting plasma insulin concentration as an estimate of insulin action in nondiabetic individuals: comparison with the homeostasis model assessment of insulin resistance (HOMA-IR). Acta Diabetol.

[32] Borai A, Livingstone C, Kaddam I, Ferns G (2011). Selection of the appropriate method for the assessment of insulin resistance. BMC Med Res Methodol.

[33] Son D-H, Lee HS, Lee Y-J, Lee J-H, Han J-H (2022). Comparison of triglyceride-glucose index and HOMA-IR for predicting prevalence and incidence of metabolic syndrome. Nutr Metab Cardiovasc Dis.

[34] Timóteo AT, Miranda F, Carmo MM, Ferreira RC (2014). Optimal cut-off value for homeostasis model assessment (HOMA) index of insulin-resistance in a population of patients admitted electively in a Portuguese cardiology ward. Acta Med Port.

[35] Tohidi M, Arbab P, Ghasemi A (2017). Assay-dependent variability of serum insulin concentrations: a comparison of eight assays. Scand J Clin Lab Invest.

