# Supplementary material for: Are HOMA-IR and HOMA-B good predictors for diabetes and pre-diabetes subtypes?
Source: BMC Endocr Disord. 2023 Feb 14;23:39. doi: 10.1186/s12902-023-01291-9 (PMC9926772; doi:10.1186/s12902-023-01291-9)
Supplement: Supplementary file 1 — Additional file 1: Table S1. Baseline characteristics of study subjects with missing and non-missing data for insulin. Table S2. Baseline characteristics of study subjects subjects who were followed and not followed. Table S3. Odds ratios of incidence of different study outcomes for HOMA-IR and HOMA-B in multiply imputed data. [file 12902_2023_1291_MOESM1_ESM.docx]

**Table S1.** Baseline characteristics of study subjects with missing and non-missing data for insulin

|  |  | Without insulin missing data (3106) | With insulin missing data (2951) | P-Value |
| --- | --- | --- | --- | --- |
| Number |  |  |  |  |
| Age (years) |  | 42.6 (11.4) | 34.9 (12.5) | <0.001 |
| Female (%) |  | 1917 (61.2) | 1596 (54.1) | <0.001 |
| Education (%) (a) | 0 | 855 (27.5) | 1020 (34.5) | <0.001 |
|  | 1 | 1785 (57.5) | 1696 (57.4) | -- |
|  | 2 | 464 (14.9) | 229 (77.6) | -- |
| Smoking (%) (b) | 0 | 2515 (80.9) | 2287 (77.5) | <0.001 |
|  | 1 | 237 (7.6) | 230 (7.8) | -- |
|  | 2 | 350 (11.2) | 430 (15.6) | -- |
| Familial history of type 2 DM (%) |  | 1075 (34.6) | 890 (30.2) | 0.846 |
| Body mass index (kg/m^2^) |  | 27.5 (4.6) | 26.3 (4.9) | <0.001 |
| Waist circumstance (cm) |  | 92.3 (11.2) | 89.6 (12.6) | <0.001 |
| Systolic Blood Pressure (mmHg) |  | 112.5 (15.4) | 109.9 (14.2) | <0.001 |
| Diastolic blood pressure (mmHg) |  | 75.9 (10.6) | 73.8 (10.3) | <0.001 |
| FBS (mmol/dl) |  | 5.0 (0.3) | 4.9 (0.3) | <0.001 |
| 2hpp BS (mmol/dl) |  | 5.3 (1.1) | 5.2 (1.1) | <0.001 |
| HDL-C (mmol/dl) |  | 1.3 (0.3) | 1.2 (0.3) | 0.015 |
| Triglyceride (mmol/dl) |  | 1.4 (0.9) | .4 (0.8) | <0.001 |
| Total cholesterol (mmol/dl) |  | 4.9 (0.9) | 4.6 (0.9) | <0.001 |

a) Education: 0 = illiterate/primary; 1 = below diploma; 2 = higher than diploma

b) Smoking: 0 = Nonsmoker; 1 = past smoker; 2 = current smoker

**Table S2.** Baseline characteristics of study subjects subjects who were followed and not followed

|  |  | Without outcome missing data (2402) | With outcome missing data (702) | P-Value |
| --- | --- | --- | --- | --- |
| Number |  |  |  |  |
| Age (years) |  | 43.1 (11.2) | 40.8 (11.6) | <0.001 |
| Female (%) |  | 1477 (61.5) | 440 (62.5) | 0.659 |
| Education (%) (a) | 0 | 643 (26.8) | 212 (30.1) | 0015 |
|  | 1 | 1378 (57.4) | 407 (57.8) | -- |
|  | 2 | 381 (15.9) | 83 (11.8) | -- |
| Smoking (%) (b) | 0 | 1957 (81.5) | 558 (79.3) | 0.017 |
|  | 1 | 191 (7.9) | 46 (6.5) | -- |
|  | 2 | 251 (10.5) | 99 (14.1) | -- |
| Familial history of type 2 DM (%) |  | 865 (36.0) | 210 (29.8) | 0.785 |
| Body mass index (kg/m^2^) |  | 27.7 (4.6) | 26.9 (4.5) | <0.001 |
| Waist circumstance (cm) |  | 92.8 (11.2) | 90.8 (10.9) | <0.001 |
| Systolic Blood Pressure (mmHg) |  | 112.7 (15.2) | 111.5 (15.8) | 0.065 |
| Diastolic blood pressure (mmHg) |  | 76.1 (10.5) | 75.1 (10.7) | 0.020 |
| FBS (mmol/dl) |  | 5.0 (0.3) | 4.9 (0.3) | <0.001 |
| 2hpp BS (mmol/dl) |  | 5.3 (1.1) | 5.2 (1.1) | 0.010 |
| HDL-C (mmol/dl) |  | 1.3 (0.3) | 1.2 (0.3) | 0.443 |
| Triglyceride (mmol/dl) |  | 1.49 (0.8) | 1.46 (0.9) | 0.049 |
| Total cholesterol (mmol/dl) |  | 4.9 9 (0.9) | 4.8 (0.9) | 0.296 |
| Fasting serum insulin (micro U/ml) |  | 8.6 (4.7) | 8.5 (4.5) | 0.423 |
| HOMA-IR |  | 1.8 (1.0) | 1.8 (0.9) | 0.238 |
| HOMA-B |  | 1.7 (0.9) | 1.7 (1.0) | 0.392 |

a) Education: 0 = illiterate/primary; 1 = below diploma; 2 = higher than diploma

b) Smoking: 0 = Nonsmoker; 1 = past smoker; 2 = current smoker

**Table S3.** Odds ratios of incidence of different study outcomes for HOMA-IR and HOMA-B in multiply imputed data

|  | iIFG | iIGT | CGI | DM |
| --- | --- | --- | --- | --- |
| HOMA-IR |  |  |  |  |
| Model-1 | 1.39 (1.26-1.53) | 1.36 (1.21-1.53) | 1.67 (1.46-1.90) | 1.76 (1.50-2.08) |
| Model-2 | 1.46 (1.33-1.60) | 1.49 (1.32-1.67) | 1.84 (1.60-2.13) | 1.86 (1.56-2.12) |
| Model-3a | 1.35 (1.20-1.53) | 1.34 (1.16-1.54) | 1.54 (1.31-1.82) | 1.69 (1.33-2.16) |
| Model-3b | 1.31 (1.18-1.46) | 1.28 (1.11-1.49) | 1.45 (1.22-1.72) | 1.50 (1.21-1.84) |
| HOMA-B |  |  |  |  |
| Model-1 | 0.99 (0.89-1.10) | 1.12 (1.01-1.25) | 1.19 (1.02-1.38) | 1.32 (1.15-1.52) |
| Model-2 | 1.07 (0.96-1.20) | 1.29 (1.16-1.43) | 1.39 (1.18-1.62) | 1.44 (1.24-1.68) |
| Model-3a | 0.96 (0.82-1.11) | 1.16 (1.03-1.30) | 1.16 (0.98-1.38) | 1.31 (1.06-1.63) |
| Model-3b | 0.91 (0.81-1.03) | 1.10 (0.99-1.24) | 1.07 (0.87-1.30) | 1.18 (0.99-1.39) |

Model (1): crude model, Model (2): Adjusted for gender and age, smoking, level of education, and family history of type 2 diabetes, Model (3a): Adjusted for model 2 in addition to metabolic syndrome variables including waist circumference, blood pressure, triglyceride, and HDL, as binary variables, model (3b): Adjusted for model 2 in addition to metabolic syndrome variables including waist circumference, systolic & diastolic blood pressure, triglyceride, and HDL as continuous variables
